# Procalcitonin (PCT) Predicts Worse Outcome in Patients with Chronic Heart Failure with Reduced Ejection Fraction (HFrEF)

**DOI:** 10.1155/2018/9542784

**Published:** 2018-08-28

**Authors:** J. Banach, Ł. Wołowiec, D. Rogowicz, L. Gackowska, I. Kubiszewska, W. Gilewski, J. Michałkiewicz, W. Sinkiewicz

**Affiliations:** ^1^2nd Department of Cardiology, Faculty of Health Sciences, Ludwik Rydygier Collegium Medicum in Bydgoszcz, Nicolaus Copernicus University, Toruń, Poland; ^2^Department of Immunology, Faculty of Pharmacy, Ludwik Rydygier Collegium Medicum in Bydgoszcz, Nicolaus Copernicus University, Toruń, Poland

## Abstract

**Introduction:**

Procalcitonin (PCT) is an excellent marker of sepsis but was not extensively studied in cardiology. The present study investigated PCT plasma concentration in patients with chronic heart failure with reduced ejection fraction (HFrEF) and its prognostic value during 24-month follow-up.

**Material and Methods:**

Study group consisted of 130 patients with HFrEF (LVEF ≤ 45%) and 32 controls. PCT level was assessed on admission in all patients. Telephone follow-up was performed every three months over a period of 2 years. Endpoints were death of all causes and readmission for HFrEF exacerbation.

**Results:**

HFrEF patients had significantly higher PCT concentration than controls (166.95 versus 22.15 pg/ml; *p* < 0.001). Individuals with peripheral oedema had increased PCT comparing to those without oedema (217.07 versus 152.12 pg/ml; *p* < 0.02). In ROC analysis, PCT turned out to be a valuable diagnostic marker of HFrEF (AUC 0.91; *p* < 0.001). Kaplan-Meier survival curves revealed that patients with PCT in the 4th quartile had significantly lower probability of survival than those with PCT in the 1st and 2nd quartiles. In univariate, but not multivariate, analysis, procalcitonin turned out to be a significant predictor of death during 24-month follow-up. (HR 1.002; 95% CI 1.000–1.003; *p* < 0.03).

**Conclusions:**

Elevated PCT concentration may serve as another predictor of worse outcome in patients with HFrEF.

## 1. Introduction

Growing population of patients with chronic heart failure is a major challenge for healthcare systems throughout the world. High rate of expensive rehospitalization is one of the main causes of economic burden associated with CHF. Risk stratification leading to identification of subgroups with the worst outcome could make the treatment process more efficient with the most aggressive strategies reserved for those with gloomy prognosis. Search for diagnostic and prognostic markers of CHF led to the discovery of the immense clinical role of human natriuretic peptides [1–3]. Haemodynamic changes occurring in failing heart resulting in natriuretic peptide synthesis are not the only one alteration in this multifaceted disease. Immunologic and inflammatory mechanisms are also affected during the natural course of chronic heart failure [4, 5]. Clinical utility of established inflammatory markers such as C-reactive protein, interleukins, and tumour necrosis factor has been extensively studied in CHF population [6–8]. Calcitonin-derived propeptide—procalcitonin—was identified in the 1990s as a marker of sepsis and invasive bacterial infection with excellent specificity and sensitivity [9]. Increased concentration of procalcitonin was reported not only in infectious diseases but also in ischemic stroke [10], in lupus exacerbation [11], and in patients with medullary thyroid carcinoma [12]. Its plausible role in cardiovascular disorders was not thoroughly investigated. Reports on procalcitonin level alterations in heart diseases are scarce and far between and are mainly focused on open-heart surgery complications. Procalcitonin is also a valuable tool in differentiating patients presenting with acute dyspnoea into those with acute heart failure and those with pneumonia [13]. Taking into account the diagnostic and prognostic significance of the traditional inflammatory markers, we decided to evaluate procalcitonin plasma level in patients with chronic heart failure with reduced ejection fraction and assess its impact on prognosis in this population.

### 1.1. Aim of the Study

The aim of the study was to investigate plasma concentration of procalcitonin in patients with chronic heart failure with reduced ejection fraction and assess its prognostic value in this population during 24-month follow-up.

## 2. Material and Methods

The study group consisted of 130 consecutive patients admitted to the university hospital department of cardiology with chronic heart failure with reduced ejection fraction. Half of them had sign or symptoms of heart failure exacerbation. The other 50% of patients were admitted electively for device implantation or periodic assessment of the disease progression. The inclusion criteria were as follows: chronic heart failure with left ventricular ejection fraction ≤ 45% assessed within past 6 months. Patients with acute coronary syndrome, acute heart failure, active infection or cancer, fever, known or suspected infection, chronic obstructive pulmonary disease, chronic inflammatory diseases, decompensated diabetes, and advanced renal insufficiency with glomerular filtration rate below 30 ml/min were excluded from the study. Clinical characteristics of the study group are summarized in Table 1.

The control group consisted of 32 healthy adult volunteers matched for age and sex. They were recruited from the outpatient cardiology department after the exclusion of cardiovascular diseases and other condition outlined in the exclusion criteria.

After explanation of the study design, each study participant signed an informed consent. Study protocol was in compliance with the principles of the Declaration of Helsinki and was approved by the Bioethical Committee of the Collegium Medicum of Nicolaus Copernicus University.

At baseline routine, laboratory tests including complete blood count, blood electrolytes, creatinine, troponin T (TnT), N-terminal brain natriuretic propeptide (NT-proBNP), and C-reactive protein (CRP) concentration were performed in each study participant. Blood samples for determination of PCT plasma concentration were collected in the supine position into Vacutainer® test tubes containing anticoagulant—3.2% sodium citrate. The blood samples were then centrifuged at 3000 ×g at 4°C for 10 minutes, and the obtained plasma was sampled into eppendorf test tubes and frozen at −80°C for no longer than 3 months before the measurement was taken. Controls' plasma samples were prepared in the same way as from CHF patients. Freeze-thaw cycles were avoided before analysis.

The remaining tests were performed in the hospital laboratory. NT-proBNP plasma level was determined by means of sandwich chemiluminescence immunoassay (Elecsys® proBNP II, Roche Diagnostics, Germany), and C-reactive protein concentration was measured with the use of high-sensitivity immunoturbidimetric method (CRP OSR6199 Highly Sensitive Application, Olympus).

PCT plasma concentration was determined in the university Department of Immunology with the use of commercially available, highly sensitive enzyme-linked immunosorbent assay (SEA 698Hu, USCN Life Science,) according to the manufacturer instructions. The intra-assay and interassay coefficients of variation were 8%–9.5% and 7.5%–9%, respectively.

All-cause mortality and hospitalization for the exacerbation of heart failure were the study endpoints. Telephone follow-up visits were performed every three months for 2 years.

### 2.1. Statistical Analysis

Statistical analyses were performed with the use of the PQStat 1.4.8 software. Kruskal-Wallis test with Bonferroni correction was used to determine the difference of the studied parameters between controls and stable and exacerbated CHF patients. Mann–Whitney *U* test was used to analyse differences in PCT plasma level between patients with or without signs of hypervolaemia. Prognostic value of PCT plasma concentration was analysed with logistic regression models. Receiver operator curves (ROC) were used to assess diagnostic significance of PCT. Kaplan-Meier survival curves were performed to explore the impact of PCT on all-cause mortality. A probability less than 0.05 was considered statistically significant.

## 3. Results

Baseline study and control group characteristic is presented in Table 1. Half of patients were admitted to the hospital with exacerbation, and half were in stable condition. Although there is no precise definition of heart failure exacerbation, we decided to include only these patients who demonstrated certain dynamics of increasing hypervolaemia ultimately urging patient to search for medical care. Low-output states were excluded from the study as in our opinion in initial stages it is difficult to make a clear distinction between plain HF exacerbation with hypotension and developing cardiogenic shock being a classical form of acute heart failure.

Plasma procalcitonin concentration was significantly higher in CHF patients than in controls. (166.95 versus 22.15 pg/ml; *p* < 0.001). Yet, no difference in PCT was recorded between exacerbated and stable study participants. There was statistically significant correlation between hsCRP level and PCT concentration (*r* 0.16; *p* < 0.05); however, no correlation between white blood cell count and PCT level was observed. PCT values together with other common inflammatory markers are presented across study groups in Table 2.

**Figure 1:**
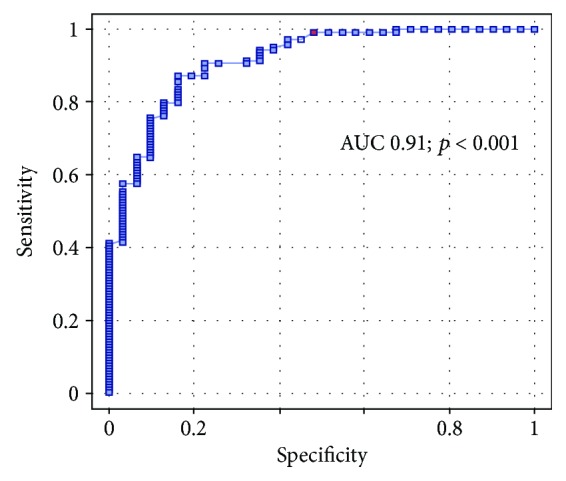
Receiver-operator curve of procalcitonin diagnostic value.

ROC analysis showed that procalcitonin is a valuable marker of chronic heart failure with reduced ejection fraction. (AUC 0.91; *p* < 0.001; Figure 1).

Moreover, PCT was also increased in individuals with peripheral oedema comparing to patients without oedema on physical examination performed on admission (217.07 versus 152.12 pg/ml; *p* < 0.02). In contrast, another sign of hypervolaemia—pulmonary congestion—did not influence PCT concentration (Table 3).

**Figure 2:**
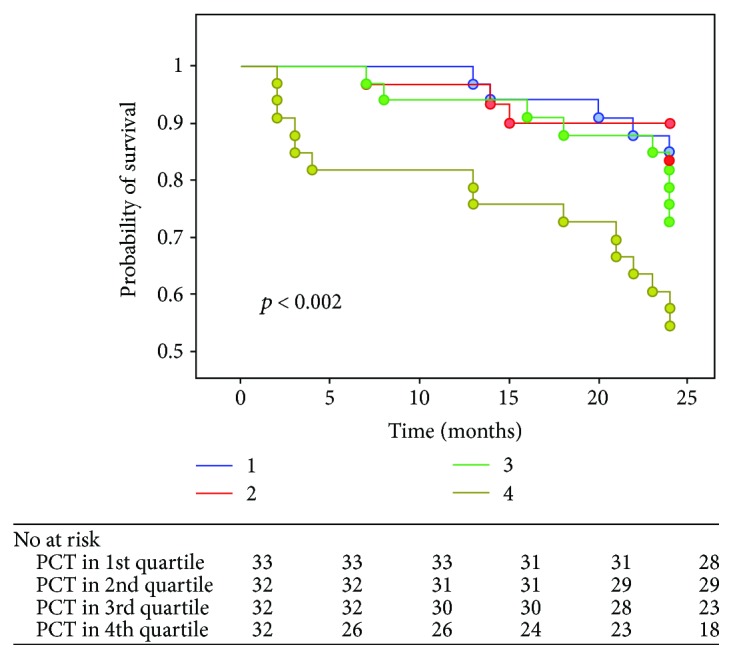
Kaplan-Meier survival curves for quartiles of PCT concentration.

In univariate analysis, procalcitonin turned out to be a significant predictor of death during 24-month follow-up (HR 1.002; 95% CI 1.000-1.003; *p* < 0.03). Kaplan-Meier survival curve highlights significant survival difference between patients with procalcitonin concentration in the 1st or 2nd and 4th quartile (*p* < 0.002) (Figure 2).

In multivariate model, only NT-proBNP (HR 1.036; 95% CI 1.008-1.0117; *p* < 0.02) and age (HR 1.061; 95% CI 1.007-1071; *p* < 0.03) independently predicted mortality. PCT plasma concentration did not allow for the prediction of the second prespecified endpoint—readmission for heart failure exacerbation.

There were statistically significant correlation between PCT and NYHA (*r* 0.3; *p* < 0.0003), NT-proBNP (*r* 0.2; *p* < 0.01) concentration, and negative correlation between PCT and LVEF (*r* − 0.17 ; *p* < 0.04).

## 4. Discussion

Similarly to our findings, Canbay et al. in a retrospective case-control study observed increased serum procalcitonin concentration in patients with chronic heart failure with LVEF < 45% comparing to controls. Moreover, individuals hospitalized with CHF had higher PCT than those in an outpatient clinic. A cut-off value set at 0.09 ng/ml (90 pg/ml) PCT allows for differentiating patients with CHF from those without the disease with all controls having negative PCT [15]. According to ROC analysis that we used to assess diagnostic accuracy of PCT, lower cut-off value of 22.8 pg/ml (99% sensitivity and 49% specificity) is optimal for diagnosing CHF. Half of our study group consisted of patients admitted to the hospital with signs of exacerbation; however, we did not observe any more pronounced elevation of procalcitonin concentration in this subgroup. After physical examination performed on admission, we also divided all patients according to signs of hypervolaemia, and those with peripheral oedema turned out to have significantly higher concentration of PCT comparing to those without oedema, but this was not the case in individuals with pulmonary congestion only. Plausible explanation of this interesting phenomenon comes from the study by Mollar et al. Authors assessed procalcitonin level together with multiple other immunologic and inflammatory markers in 261 patients diagnosed with the episode of acute heart failure after exclusion of active infection in an emergency department setting. Significant correlation was found between PCT concentration and surrogate markers of inflammation including leukocyte count, low lymphocyte number, low high-density lipoproteins, and endotoxin level. Moreover, signs of venous congestion such as sodium and gamma-glutamyl transpeptidase level and low estimated glomerular filtration rate turned out to increase PCT concentration. Authors conclude that all the evidence points out toward the endotoxin stimulation resulting from bacterial translocation through the intestine wall in patients with peripheral congestion. Altered intestine function leading to the stimulation of inflammatory process in patients with heart failure is not a new concept in pathophysiology of this multifactorial syndrome [16]. The most recent publication by Pasini et al. proves that not only intestine permeability is increased in CHF patients but also massive bacterial and fungal overgrowth can be observed in this population. Apart from microbiologic investigation and intestinal barrier properties evaluation, Pasini et al. assessed also right atrial pressure (RAP) as a surrogate marker of congestion and C-reactive protein as the most common marker of systemic inflammation. The observed positive correlation between increased intestine permeability and RAP is in concordance with our findings of higher procalcitonin level in patients with peripheral oedema [17]. All together, these findings provide plausible mechanistic explanation for the observed CHF-associated increase of procalcitonin. Yet, we did not find any difference in terms of CRP concentration between patients with or without peripheral oedema. In our opinion, this observation indicates that PCT with its high sensitivity and specificity in diagnosis of bacterial bloodstream infection may be a more valuable indicator of bacterial translocation associated with advanced heart failure than such a versatile marker as CRP. Another observation confirming that heart failure may be a reason behind PCT elevation comes from a relatively recent study by Wang et al. Authors in a cohort of 4698 patients with dyspnoea recorded higher PCT concentration in patients with heart failure than in controls together with higher PCT in CHF complicated by the infection comparing to patients with infection only. These results indicate the significant diagnostic value of PCT in differential diagnosis of patients with dyspnoea managed in an emergency setting [18].

In our study group, PCT, in univariable model, turned out to significantly impact all-cause mortality during 24-month observation. Patients with PCT in the 4th quartile had significantly lower chance of survival than those in the 1st and 2nd quartiles. Prognostic value of procalcitonin concentration has been described previously, however, only in patients with acute heart failure (AHF). Loncar et al. evaluated the impact of both baseline procalcitonin level and the increase of PCT concentration during hospitalization in patients admitted with AHF episode. Persistent elevation of PCT as well as its increase during the first 72 hours of hospitalization was associated with the worst outcome during 3-month follow-up [19]. Similar findings come from BACH trial in which PCT turned out to predict worse outcome not only in patients with pneumonia but also in individuals with acute heart failure. BACH investigators underscore that PCT is not only a valuable addition to routine procedures used in initial diagnosis in patients presenting with dyspnoea but also provides prognostic information and can help to choose treatment modality [13]. The most recent study addressing PCT's role in predicting outcome in patients with heart failure also focuses on cases of acute heart failure. Villanueva et al. followed 261 consecutive patients admitted with the diagnosis of AHF without signs of infection in whom baseline PCT was determined. PCT accurately predicted both death of all causes and rehospitalization. In our research, we did not record the influence of PCT concentration on readmission; however, the length of observation—considerably shorter in our study—might be the reason behind the lack of the relationship between PCT and this endpoint [20].

## 5. Conclusions

The present study is to the best of our knowledge the first research on procalcitonin diagnostic and prognostic value in chronic heart failure population. Following promising results of trials involving patients with AHF, this study provides another set of evidence on the emerging role of procalcitonin in heart failure diagnostic process and risk stratification. Since the incorporation of natriuretic peptide assessment into routine clinical practice, numerous plausible markers of heart failure have been tested with varying results. Taking into account the high residual risk reflecting multifaceted pathophysiology of heart failure, natriuretic peptides even with their immense clinical role seem to be insufficient. Thus, multimarker approach may help to refine therapeutic strategies and allow for tailored treatment adjusted to the individual clinical and biochemical profile of each heart failure patient.

## Figures and Tables

**Table 1:** Clinical and laboratory characteristics of stable and exacerbated patients [14].

	All	Stable	Exacerbated	*p*
Clinical and demographic indices				
*n*	130	65	65	—
Males	107	55	52	ns
Age (years; mean ± SD)	59.8 ± 13.1	57.3 ± 9.8	61.5 ± 14.1	ns
BMI (kg/m^2^; mean ± SD)	28.7 ± 5.8	28.2 ± 5.6	28.6 ± 6.0	ns
Ischemic aetiology	66 (53%)	32 (49%)	34 (56%)	ns
NYHA II/III/IV	39%/51%/9% 52/66/12	55%/45%/0% 36/29/0	24%/57%/18% 16/37/12	<0.001^∗^
Peripheral oedema	40 (31%)	8 (12%)	32 (49%)	<0.05^∗^
Pulmonary congestion	37 (28%)	5 (8%)	32 (49%)	<0.05^∗^
LVEF (%; mean ± SD)	27 ± 8	27 ± 7	26 ± 8	ns
DM	47 (36%)	21 (32%)	26 (40%)	ns
Smoking	37 (28)	19 (25%)	18 (24%)	ns
Death	32 (25%)	12 (18%)	20 (30%)	<0.001^∗^
Hospitalization	60 (46%)	33 (51%)	27 (41%)	ns
Laboratory tests				
Hb [g/dl]	13.9 ± 1.5	14.1 ± 0.9	13.7 ± 1.9	ns
Nt-proBNP [pg/ml]	1862 ± 5957	1643.92 ± 1776	5142 ± 7391	<0.0001^∗^
Troponin T [*μ*g/l]	0.02 ± 0.03	0.03 ± 0.03	0.03 ± 0.02	ns
Treatment				
ACEI	105 (82%)	51 (78%)	54 (83)	ns
ARB	23 (18%)	14 (22%)	9 (14%)	ns
Betablockers	129 (99%)	65 (100%)	64 (99%)	ns
Statin	105 (81%)	58 (89%)	47 (72%)	ns
ASA	61 (47%)	26 (40%)	35 (54%)	ns
Digoxin	35 (27%)	22 (34%)	13 (20%)	<0.05^∗^
Spironolactone	96 (74%)	45 (69%)	51 (78%)	ns
Eplerenone	23 (18%)	15 (23%)	8 (12%)	<0.05^∗^
Diuretic	101 (78%)	36 (55%)	65 (100%)	<0.05^∗^

BMI: body mass index; NYHA: New York Heart Association; LVEF: left ventricular ejection fraction; DM: diabetes mellitus; Hb: haemoglobin; NT-proBNP: N-terminal probrain natriuretic peptide; ACEI: angiotensin converting enzyme inhibitors; ARB: angiotensin receptor blockers; ASA: acetylsalicylic acid. ^∗^*p* value for the difference between stable and exacerbated patients.

**Table 2:** Comparison of white blood cell count (WBC), procalcitonin (PCT), and high sensitivity C-reactive protein (hsCRP) plasma concentration in patients with chronic heart failure: stable and exacerbated and in controls.

Group	Minimum	Lower quartile	Median	Upper quartile	Maximum	*p*
Procalcitonin [pg/ml]						
Control	1.19	13.00	22.15	48.74	200	<0.0001^∗^
CHF all	14.71	112.27	166.95	284.53	927	
CHF stable	14.71	118.65	163.71	280.47	800.24	
CHF exacerbation	22.83	105.46	175.19	286.54	927.00	
hsCRP [mg/l]						
Control	0.3	1.7	2.2	2.5	6.6	<0.01^∗^
CHF all	0.6	1.9	4.0	7.8	48	
CHF stable	0.6	1.6	3.4	7.2	23.7	
CHF exacerbation	0.8	2.1	4.6	8.2	48	
WBC [G/l]						
Control	4.61	5.70	6.27	8.87	9.18	ns
CHF all	4.03	5.98	7.32	11.12	13.74	
CHF stable	4.83	6.10	7.28	11.23	13.74	
CHF exacerbation	4.03	5.65	7.37	10.91	12.82	

^∗^Difference between CHF all and controls.

**Table 3:** Procalcitonin in patients with or without pulmonary congestion and peripheral oedema.

Group	Minimum	Lower quartile	Median	Upper quartile	Maximum	Mann–Whitney *U* test
Procalcitonin [pg/ml]						
Oedema	22.83	146.4	217.07	355.91	787.36	*p* = 0.02
No oedema	14.71	99.5	152.12	246.76	927.00	
Congestion	22.83	122.4	207.47	356.88	927.00	*p* = 0.07
No congestion	15.71	108.18	153.56	234.32	787.36	

## Data Availability

All data used to support the finding of this study are available from the corresponding author upon request.
